# Long-term spatiotemporal dynamics of β-lactamase resistome in the Han River, South Korea

**DOI:** 10.3389/fmicb.2026.1903212

**Published:** 2026-10-02

**Authors:** Do-Hoon Lee, Yong-Seok Kim, Dae-Wi Kim, Dong-Sook Kim, Chang-Jun Cha

**Affiliations:** 1 Department of Systems Biotechnology and Center for Antibiotic Resistome, Chung-Ang University, Anseong, Republic of Korea; 2 Department of Life Sciences, Jeonbuk National University, Jeonju, Republic of Korea; 3 Department of Health Administration, College of Health, Kongju National University, Gongju, Republic of Korea

**Keywords:** antimicrobial resistance, aquatic environments, β-lactamases, environmental resistome, spatiotemporal dynamic

## Abstract

The global dissemination of antimicrobial resistance, particularly resistance to widely prescribed β-lactam antibiotics, is a critical public health threat worldwide. Aquatic ecosystems act as key reservoirs where antibiotic resistance genes accumulate and are transmitted between environmental bacteria and human pathogens within a One Health framework. To move beyond short-term observations and better understand these cross-sectoral dynamics, we conducted 5-year longitudinal surveillance using shotgun metagenomics across different seasons along the Han River, South Korea. Our analysis revealed a diverse repertoire of β-lactamases, predominantly belonging to Ambler classes A and D, which also accounted for most mobile β-lactamase genes and were enriched in densely populated downstream regions. Furthermore, β-lactamase genes exhibited clear seasonal fluctuations, with a pronounced winter surge in total and mobile β-lactamase gene abundances. We also identified a substantial fraction of novel variants, whose abundance and composition varied across spatial and seasonal gradients. Network analysis revealed that the genera *Pseudomonas* and *Klebsiella* acted as primary hosts, with their mobile β-lactamase genes highly connected to multiple taxa, suggesting important roles in β-lactamase gene transmission. These findings suggest that preemptive management of aquatic β-lactamase genes is a fundamental strategy for mitigating future resistance crises within a One Health framework and provide critical evidence for identifying transmission pathways and tracking resistance evolution between natural and clinical environments.

## 1 Introduction

Antimicrobial resistance (AMR) is widely recognized as a “silent pandemic” and the dissemination of AMR stands as one of the most critical threats to global public health (Mendelson et al., 2022; Burki, 2023). Historically, AMR research has predominantly focused on human pathogens in clinical settings. However, the One Health paradigm has reframed this narrow perspective by highlighting that human, animal, and environmental health are deeply interconnected (Kim and Cha, 2021; Li et al., 2025). Within this holistic framework, it has become evident that natural environments, particularly aquatic ecosystems, serve as massive reservoirs and hotspots that drive the evolution and dissemination of antibiotic resistance genes (ARGs) (Baquero et al., 2008; Finley et al., 2013). Various anthropogenic sources, including wastewater treatment plants (WWTPs), agricultural runoff, and hospital and domestic sewage, introduce contaminants into aquatic environments (Ehalt Macedo et al., 2025). Such pollutants exert selective pressure on aquatic microbial communities and facilitate horizontal gene transfer, creating a feedback loop in which ARGs are repeatedly exchanged between environmental bacteria and human pathogens (Larsson and Flach, 2022).

Among diverse ARGs, genes encoding β-lactamases, which confer resistance to β-lactam antibiotics, are regarded as some of the most clinically important and environmentally concerning resistance determinants. β-Lactam antibiotics, including *penicillins*, *monobactams*, *cephalosporins*, and *carbapenems*, are among the most frequently prescribed essential drugs in clinical settings worldwide (Wang et al., 2025). In particular, extended-spectrum β-lactamases (ESBLs; e.g., *bla*_CTX–M_, *bla*_TEM_, and *bla*_SHV_), which inactivate a broad range of β-lactams, and carbapenemases (e.g., *bla*_NDM_, *bla*_KPC_, and *bla*_OXA_), which confer resistance to carbapenems, are major obstacles to successful antimicrobial therapy in clinical practice (Tooke et al., 2019). Furthermore, the dissemination of β-lactamases via mobile genetic elements (MGEs), including plasmids, integrons, and transposons, has been widely reported (Tooke et al., 2019). The frequent detection of β-lactamases in effluents from WWTPs (Makowska et al., 2020), rivers (Lee et al., 2020), and even polar aquatic environments (Bisaccia et al., 2025) suggests that the spread of AMR is proceeding on a global scale and is a natural and ubiquitous phenomenon that transcends the boundaries of clinical, veterinary, and environmental domains. Consequently, relying solely on studies confined to clinical settings leads to an incomplete understanding of the complex AMR problem.

Despite rapid advances in sequencing technologies and a growing consensus on the necessity of the One Health approach, most environmental resistome studies still focus on simple descriptive reporting of phenomena in environmental samples. Such descriptive approaches have clear limitations in elucidating the complex evolutionary processes and cross-sectoral dynamics underlying AMR. Furthermore, although the aquatic resistome is strongly shaped by temporal and seasonal variability, most existing studies rely on fragmented spatial snapshots or short-term monitoring, which constrains the assessment of long-term evolutionary trajectories and seasonal periodicity.

To address these limitations, the present study investigates the spatiotemporal dynamics and evolutionary patterns of the β-lactamase resistome using longitudinal metagenomic samples collected over a 5-year period from the Han River, South Korea. This large-scale riverine system is exposed to varying degrees of anthropogenic influence, ranging from relatively pristine upstream areas to heavily urbanized downstream regions. By analyzing a highly structured time series of samples collected annually in February, May, and August (or September), our study quantitatively evaluates the effects of temporal and seasonal variability on the dissemination of the environmental β-lactamase resistome. The metagenomic dataset analyzed in this study is the same longitudinal riverine dataset used in a separate manuscript focusing on the entire resistome in the Han River. In contrast, the present work provides an in-depth, β-lactamase–focused analysis, including lineage-level dynamics and novel variant characterization, which were not examined in the previous study.

## 2 Materials and methods

### 2.1 Sample collection and shotgun metagenomic sequencing

Surface water samples were collected from 15 distinct sites along the Han River (Supplementary Figure 1 and Supplementary Table 1). Sampling was conducted from May 2016 to August 2020, encompassing three different seasons: spring (May), summer (August), and winter (February). Details of the sampling methodology are described in our previous study (Lee et al., 2020). Environmental DNA extraction was performed utilizing the DNeasy PowerWater Kit (QIAGEN, Hilden, Germany) according to the manufacturer’s instruction. Shotgun sequencing libraries were prepared using the Illumina TruSeq Nano DNA kit (San Diego, CA, USA) according to the manufacturer’s protocol. Subsequently, these libraries were sequenced via 151-bp paired-end sequencing on an Illumina HiSeq 4000 platform (Macrogen, Seoul, Korea). One sample from site H7 collected in summer 2018 was excluded because repeated sequencing attempts were unsuccessful.

### 2.2 Assembly and taxonomic classification

Raw reads of shotgun metagenome sequencing were pre-processed to remove adapter sequences and filter out low-quality reads using FaQCs. Metagenomic assembly was executed using IDBA-UD v1.1.3 (Peng et al., 2012). To estimate contig abundance, the pre-processed reads were mapped to the metagenome contigs using Bowtie2 v2.4.0 (Langmead and Salzberg, 2012), and the average coverage depth for each metagenome contig was calculated using SAMtools v1.15.1 (Li et al., 2009). Taxonomic assignment of metagenome contigs was performed using Kraken v2.0 (Wood et al., 2019) with default settings, utilizing the standard Kraken2 database comprising bacterial, archaeal, and viral genomes. Open reading frames (ORFs) within metagenome contigs (≥500 bp) were predicted using Prodigal v2.6.3 (Hyatt et al., 2010) under default parameters with the addition of the “-p meta” option.

### 2.3 Collection of β-lactamase sequences

A total of 11,569 β-lactamase sequences were retrieved from the β-lactamase database (BLDB) (Naas et al., 2017) to serve as a reference for identifying β-lactamase sequences within metagenome contigs and bacterial genomes. For the metagenome contigs, the predicted ORFs were annotated using blastp function in DIAMOND v2.0.15 (Buchfink et al., 2015) with maximum e-value 1 × 10^–10^, and 90% identity, and 50% subject coverage. Similarly, the ORFs for the bacterial genome sequences (*n* = 303,784) obtained from the NCBI RefSeq database were predicted using Prodigal v2.6.3 and annotated β-lactamase using DIAMOND v2.0.15. To extract non-redundant sequences, the collected β-lactamase sequences were clustered at 99% identity and 90% coverage using MMseqs2 v14.7e284 (Steinegger and Soding, 2017). These non-redundant sequences were employed to build a network to identify shared genes between bacterial hosts and the β-lactam resistome in riverine environments. Bipartite network modules were computed using Beckett’s algorithm in the bipartite package, followed by network visualization using a Fruchterman-Reingold layout in the igraph package in R.

### 2.4 Quantification and normalization of β-lactamase gene abundance

β-Lactamase gene abundance was quantified using contig coverage depth. Briefly, the mean per-base coverage depth of each contig was calculated by dividing the total sequencing depth across all bases in a contig by its length. For each of 40 universal single-copy phylogenetic marker genes (SCGs), the coverage depths of all contigs carrying it in the sample were calculated. The mean coverage depth of each contig carrying β-lactamase gene was then divided by the median coverage depth of 40 SCGs, yielding the relative abundance of β-lactamase genes expressed as copies per genome equivalent (Equation 1).


$$A_{g,s}=\frac{C_{c\,\left(g\right),s}}{m\,e\,d\,i\,a\,n\,\left(C_{S\,C\,G_{1},s},\ldots ,C_{S\,C\,G_{40},s}\right)}$$ (1)

where *A*_*g,s*_ is the normalized abundance of β-lactamase gene *g* in sample *s*, *C*_*c(g),s*_ is the mean coverage depth of the contig carrying gene *g*, and the denominator is the median coverage depth of the 40 SCGs in the same sample.

### 2.5 Mobility analysis of β-lactamases

Mobile β-lactamase genes were identified as previously described with some modifications (Lee et al., 2023), based on the presence of identical sequences across multiple bacterial species determined via clustering or the localization on a plasmid-like contig. Briefly, the β-lactamase sequences identified from the Han River samples were pooled with 303,784 NCBI RefSeq genomes (filtered to genomes with ≤100 contigs) and clustered using MMseqs2 at a 99% identity and 90% coverage threshold. Clusters containing β-lactamase sequences of only a single species origin were classified as non-mobile β-lactamase genes, whereas those spanning multiple species were classified as mobile β-lactamase genes. In addition, if ARGs are carried in plasmid-like contigs, those β-lactamase genes are also considered mobile.

Identification of plasmid-like contigs in the metagenomic contigs was performed using five different methods. First, 39,967 plasmid sequences were downloaded from the NCBI RefSeq database^1^ and utilized to screen for plasmid-like contigs via blastn implemented in standalone BLAST+ programs (McGinnis and Madden, 2004). Metagenomic contigs were classified as candidate plasmid-like contigs if they exhibited at least 90% identity with an alignment length of at least 1,000 bp. Second, deep learning-based software tools, such as PlasClass (Pellow et al., 2020), PlasFlow (Krawczyk et al., 2018), PlasForest (Pradier et al., 2021), and PlasmidNet (Lee et al., 2023), were employed with default parameters to identify candidate plasmid-like contigs. Finally, a metagenome contig was defined as a plasmid-like contig if it was identified as such by at least three of the five methods.

### 2.6 Statistical analysis

All statistical analyses were conducted using R (R Core Team, 2022). Differences in β-lactamase gene abundance across geographic locations, seasons, mobility, and variant groups were evaluated using the Wilcoxon rank-sum test or Kruskal-Wallis test, as appropriate. When multiple *post-hoc* pairwise comparisons were performed, *p*-values were adjusted using the Bonferroni correction. α-Diversity was assessed using the Shannon diversity index. To determine significant variations in the composition of β-lactamase genes, analysis of similarities (ANOSIM) was conducted based on Bray-Curtis distance matrices. Both Shannon index calculation and ANOSIM were performed using the *vegan* package (Oksanen et al., 2024). Temporal trends were analyzed using a generalized linear model (GLM).

## 3 Results

### 3.1 β-lactamase repertoire of the Han River

Metagenomic analysis of 209 freshwater samples from the Han River identified a total of 500 β-lactamase sequences (Supplementary Data Sheet 1). Phylogenetic analysis of their amino acid sequences revealed a high degree of diversity among these riverine β-lactamases. Ambler classes A (*n* = 245) and D (*n* = 218) were the most prevalent, whereas classes B (*n* = 19) and C (*n* = 18) were detected at substantially lower frequencies (Figures 1A,B). Among the 32 distinct β-lactamase families identified, the OXA family was predominant (*n* = 199), followed by the GES (*n* = 92), GPA (*n* = 52), and PAU (*n* = 48) families (Figures 1A,B). In contrast, Ambler classes B and C were represented exclusively by low-abundance families comprising three or fewer members each (Figure 1B). Taxonomic assignment revealed that the genera *Pseudomonas*, *Aeromonas*, and *Klebsiella* were the primary hosts for these environmental β-lactamases. *Aeromonas*, *Stenotrophomonas*, *Acinetobacter*, and *Elizabethkingia* were found to harbor three or more β-lactamase classes, whereas other genera possessed only class A or both class A and class B β-lactamases (Figure 1C). Among the major families, OXA β-lactamases exhibited the broadest host range (Supplementary Figure 2). The GES, GPA, and ORF6 families were distributed across at least three different genera, whereas each of the other families was restricted to a single genus (Supplementary Figure 2).

**FIGURE 1:**
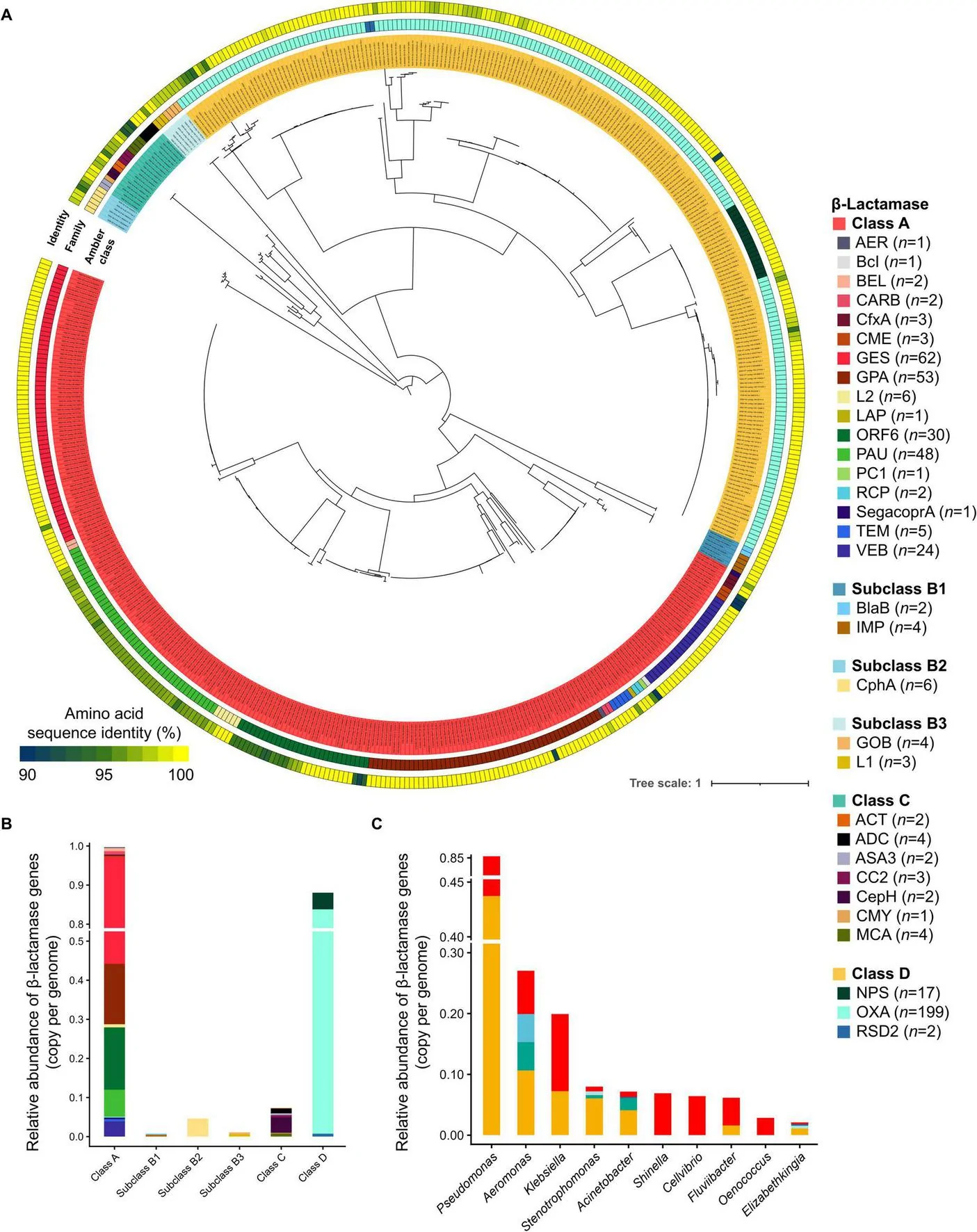
Diversity and distribution of #x03B2;-lactamases in the Han River. **(A)** Phylogenetic tree of β-lactamase amino acid sequences. Shading colors on sequence names indicate the Ambler class of each β-lactamase. Colors in the inner circle represent β-lactamase families, whereas colors in the outer circle indicate amino acid sequence identity to the closest β-lactamase reference sequence in the BLDB. **(B)** Distribution of identified β-lactamase Amble classes and families. **(C)** Distribution of Ambler classes of identified β-lactamase sequences across major bacterial genera.

### 3.2 Mobile vs. non-mobile β-lactamase genes

Given this repertoire, we next examined the mobility of β-lactamase genes by classifying them into mobile and non-mobile groups (Methods 2.5). Among the detected β-lactamase genes, 74.5% were categorized as mobile and 25.5% as non-mobile. The abundance of mobile β-lactamase genes was significantly higher than that of non-mobile β-lactamase genes (Wilcoxon rank-sum test, *p* = 1.83 × 10^–15^; Figure 2A). While non-mobile β-lactamase genes were distributed relatively evenly across all Ambler classes, mobile β-lactamase genes were dominated by classes A and D (Figure 2B). Mobile β-lactamase genes were detected in 18 of 32 families identified in the Han River; all genes assigned to the CARB, CfxA, GES, TEM, VEB, IMP, CMY, and MCA families were exclusively mobile, whereas GPA, ORF6, and OXA families were predominantly mobile (Figure 2C). In contrast, AER, Bcl, CME, L2, LAP, PC1, SegacoprA, BlaB, CphA, GOB, L1, ADC, ASA3, and CepH families were exclusively non-mobile.

**FIGURE 2:**
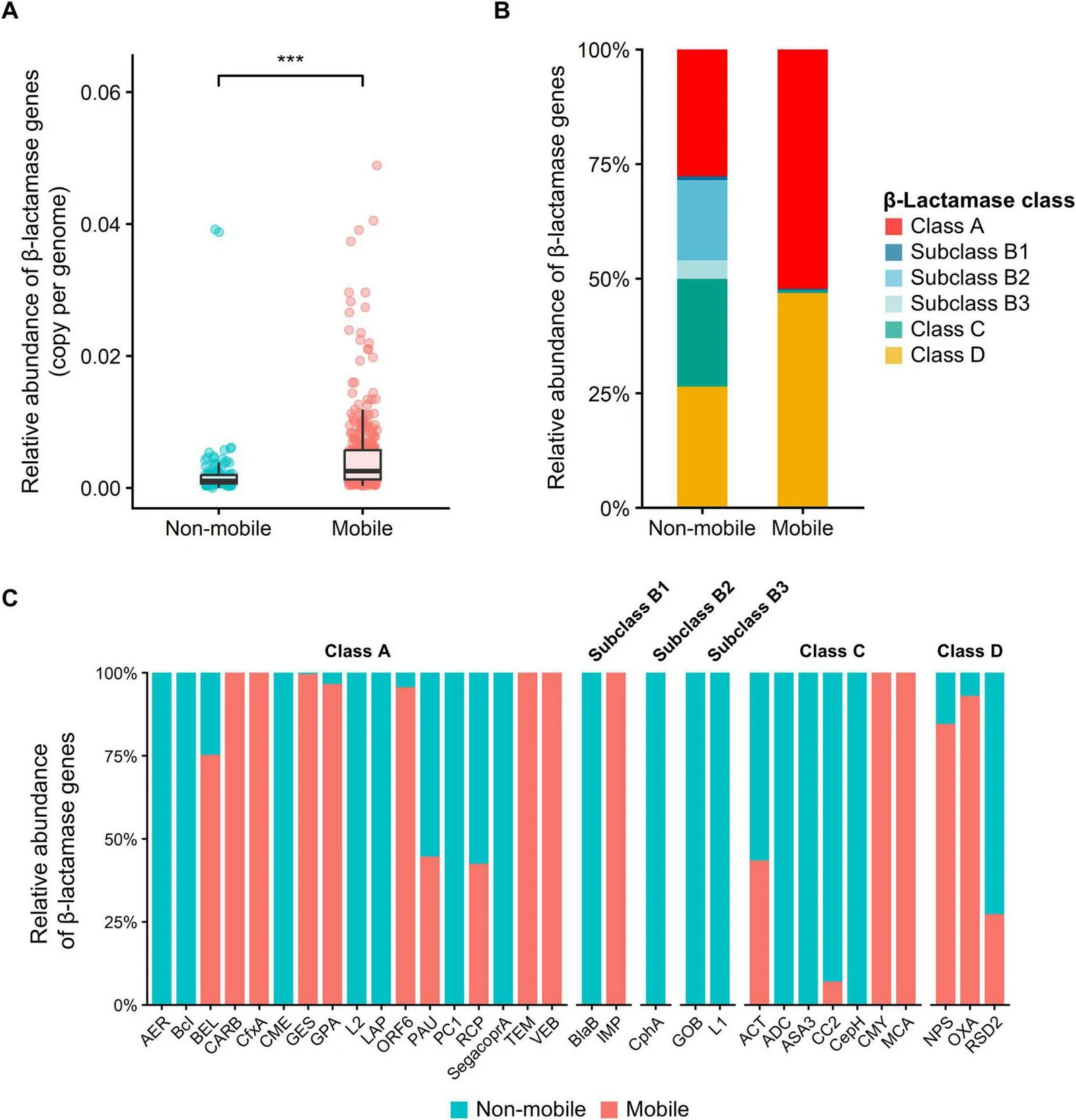
Characterization of mobile vs. non-mobile β-lactamase genes. **(A)** Comparison of β-lactamase gene abundance between mobile and non-mobile groups. Statistical significance is indicated by *** (*p* < 0.001). **(B)** Composition of β-lactamase Ambler classes according to gene mobility. **(C)** Distribution of mobile and non-mobile β-lactamase genes across different β-lactamase families.

### 3.3 Geographical distribution of β-lactamase

To investigate the geographical distribution of β-lactamase genes, sampling sites were categorized into upstream and downstream regions, with the highly populated metropolitan area (H2–H7) defined as the downstream region (Supplementary Figure 1 and Supplementary Table 1). The downstream enrichment of β-lactamases in the Han River was particularly pronounced by classes A and D (Figures 3A,B). The overall abundance of the β-lactamases in the Han River was significantly higher downstream than upstream (Wilcoxon rank-sum test, *p* = 1.92 × 10^–12^; Figure 3C). Geographic comparison of Shannon index revealed that β-lactamase α-diversity was significantly higher in downstream region than in upstream region (Wilcoxon rank-sum test, *p* = 3.72 × 10^–11^; Figure 3D). β-Diversity analysis evaluating the differences in β-lactamase composition among samples revealed distinct clustering patterns and the compositional profiles differed significantly between upstream and downstream locations (ANOSIM, *R* = 0.435, *p* = 0.001; Figure 3E). Spatial comparisons of β-lactamase gene mobility revealed no statistically significant differences in relative abundance between the mobile and non-mobile groups in the upstream region (Figure 3F). In contrast, the mobile β-lactamase group was significantly more abundant than the non-mobile group in the downstream region (Wilcoxon rank-sum test, *p* = 2.32 × 10^–11^; Figure 3F). Specifically, the non-mobile PAU and the mobile OXA, PAU, and GES families exhibited significant increases in the downstream region (Figure 3G).

**FIGURE 3:**
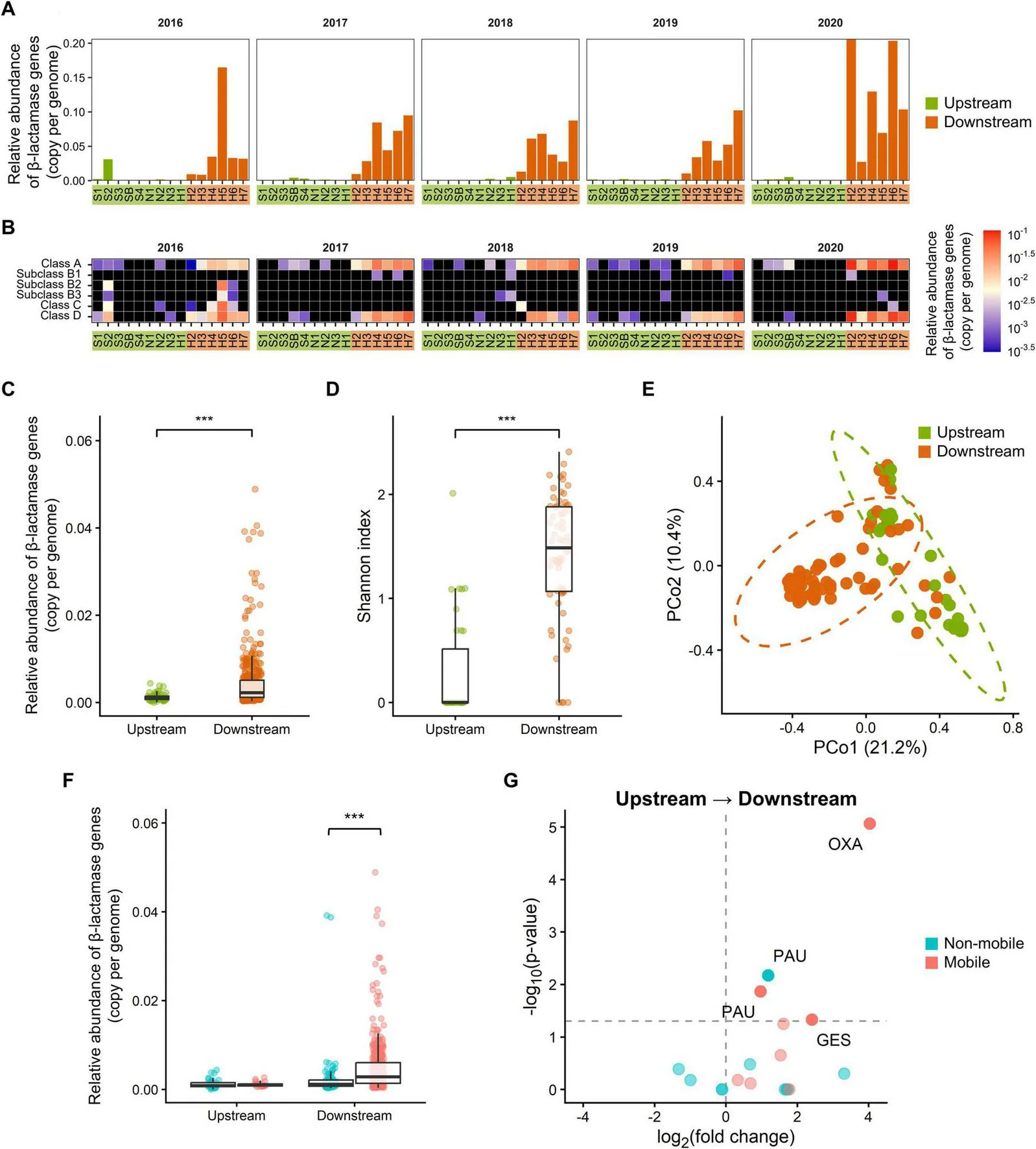
Geographical distribution and diversity of β-lactamase genes. **(A)** β-Lactamase gene abundance across sampling sites over the study period. **(B)** Heatmaps showing β-lactamase gene abundance according to Ambler classes. **(C)** Comparison of β-lactamase gene abundance between upstream and downstream regions. **(D)** Comparison of α-diversity (Shannon index) of β-lactamase genes between upstream and downstream regions. **(E)** Principal coordinate analysis (PCoA) of β-diversity based on the Bray–Curtis dissimilarity matrix of β-lactamase profiles. **(F)** Comparison of β-lactamase gene abundance between mobile and non-mobile groups in upstream and downstream regions. **(G)** Differences in the abundance of mobile and non-mobile β-lactamase genes along the upstream-to-downstream gradient. Statistical significance is indicated by *** (*p* < 0.001).

### 3.4 Temporal and seasonal dynamics of β-lactamases in the Han River

Long-term temporal analysis over the 5-year period revealed that the relative abundance of β-lactamase genes increased significantly (GLM, slope = 6.04 × 10^–5^, *p* = 5.32 × 10^–5^; Figure 4 and Supplementary Table 2). Ambler classes A and D, which account for the majority of β-lactamase genes, also demonstrated significantly increasing trends over time (GLM, slope = 7.62 × 10^–5^, *p* = 0.0002 for class A, slope = 6.41 × 10^–5^, *p* = 0.0042 for class D; Figure 4 and Supplementary Table 2).

**FIGURE 4:**
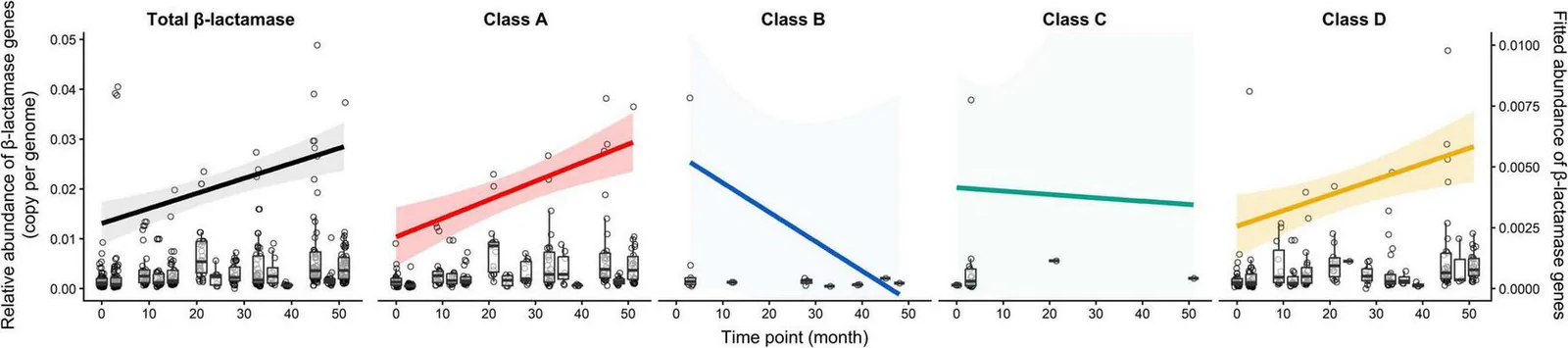
Long-term temporal dynamics of β-lactamase genes across Ambler classes. Linear trend lines were determined using a generalized linear model (GLM), and shaded areas indicate 95% confidence intervals. The secondary y-axis on the right corresponds to the GLM-fitted trends and their confidence intervals.

β-Lactamase gene abundance also exhibited pronounced seasonal patterns. Downstream enrichment of β-lactamase genes was observed across all seasons (Supplementary Figure 3A), and their relative abundance was significantly higher in winter than in the other seasons (Kruskal-Wallis test followed by Dunn’s *post-hoc* test, *p* < 0.001; Figure 5A). While the Shannon index showed no significant seasonal difference in α-diversity (Supplementary Figure 3B), a distinct seasonal pattern was observed for β-diversity (ANOSIM, *R* = 0.130, *p* = 0.001; Supplementary Figure 3C). Seasonal comparisons based on β-lactamase gene mobility revealed significantly higher levels of mobile β-lactamase genes than non-mobile β-lactamase genes across all seasons (Wilcoxon rank-sum test, *p* < 0.001; Figure 5B). Analysis of trends in β-lactamase gene abundance across seasonal transitions revealed that both total and mobile β-lactamase gene abundances decreased significantly during the winter-to-spring transition, but increased significantly during the spring-to-summer and summer-to-winter transitions (Figure 5C, Supplementary Figures 4A,B, and Supplementary Table 3). In contrast, non-mobile β-lactamase genes showed a significantly decreasing trend during the winter-to-spring transition, whereas no significant changes were observed during the other seasonal transitions (Figure 5C, Supplementary Figure 4C, and Supplementary Table 3). β-Lactamase family-level analysis across seasonal transitions revealed that non-mobile OXA and mobile GES, VEB, and OXA families decreased significantly during the winter-to-spring transition (Figure 5D), whereas the mobile VEB family increased significantly during the spring-to-summer transition and the mobile GES family was enriched during the summer-to-winter transition. These results suggest that seasonal fluctuations in β-lactamase abundance in the Han River, particularly the winter peak, are primarily driven by mobile β-lactamase genes.

**FIGURE 5:**
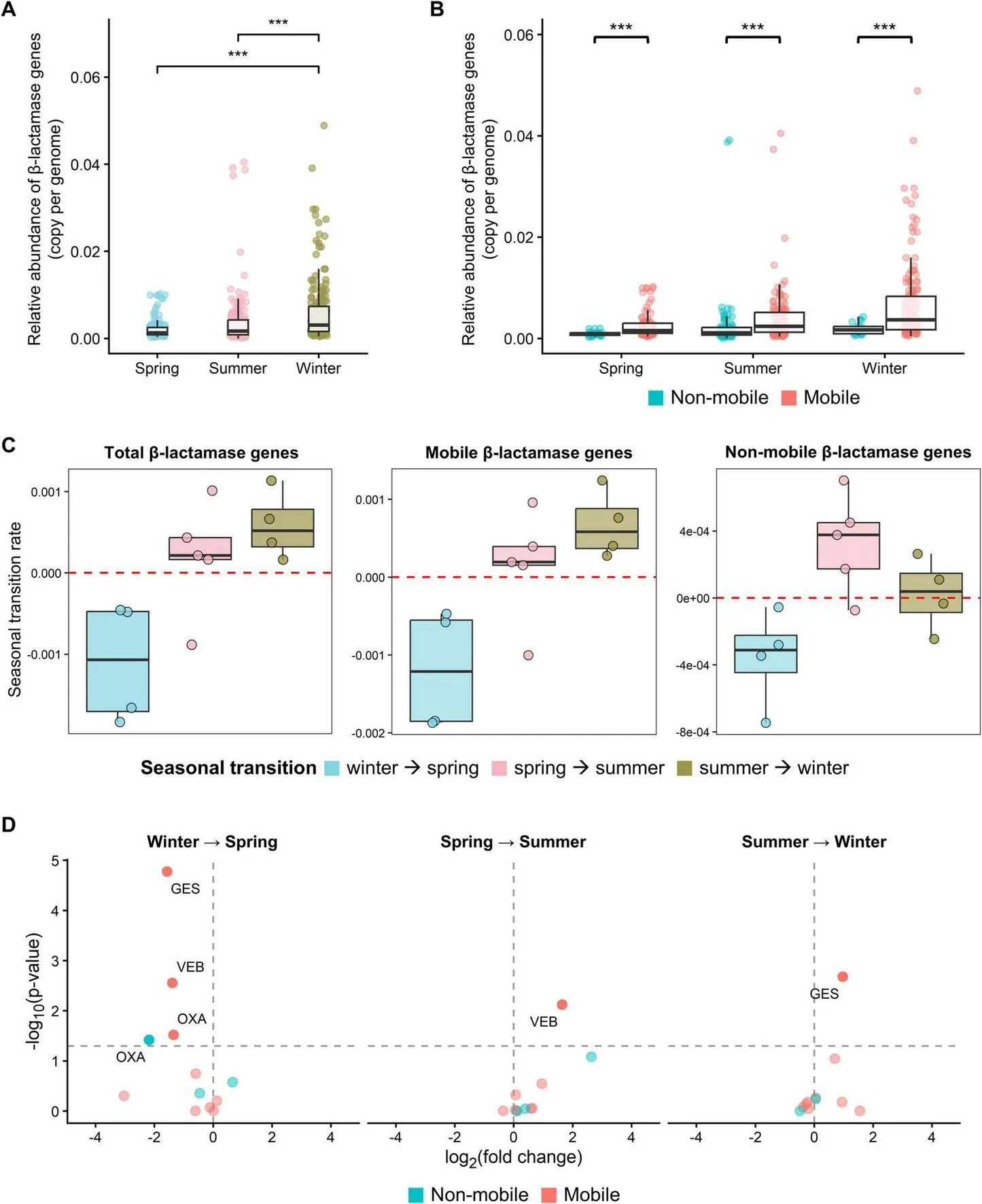
Seasonal dynamic of β-lactamase genes. **(A)** Comparison of β-lactamase gene abundance across seasons. **(B)** Comparison of β-lactamase gene abundance between mobile and non-mobile groups across seasons. Statistical significance is indicated by *** (*p* < 0.001). **(C)** Inter-seasonal transition rates (GLM slopes) of total, mobile, and non-mobile β-lactamase gene abundances. Rates represent proportional changes in β-lactamase gene abundance between consecutive seasons across the study period. **(D)** Differences in the abundance of mobile and non-mobile β-lactamase gene families according to seasonal transitions.

### 3.5 Prevalence of novel β-lactamase variants

Of the 500 β-lactamase genes detected in the Han River, 312 were identical to sequences reported in the BLDB, whereas 188 exhibited at least one amino acid substitution. The 188 β-lactamase sequences identified across different contigs represented 151 unique novel β-lactamase variants based on their amino acid sequences, in accordance with standard β-lactamase nomenclature (Figure 6A and Supplementary Figure 5). The OXA family harbored the highest number of novel variants (*n* = 75) and displayed a broad range of amino acid substitutions, followed by the PAU (*n* = 45) and ORF6 (*n* = 19) families (Figure 6A and Supplementary Figure 5). Significant differences in relative abundance between reported and novel variants were observed in both the upstream and downstream regions (Wilcoxon rank-sum test, *p* = 0.0200 for upstream; *p* = 3.36 × 10^–5^ for downstream; Figure 6B). Seasonal analysis also revealed significant differences in relative abundance between the two variant groups in spring (Wilcoxon rank-sum test, *p* = 1.35 × 10^–5^) and summer (*p* = 0.0001), whereas no significant difference was observed in winter (*p* = 0.1009; Figure 6B). Interestingly, contrasting patterns were observed according to mobility of β-lactamase genes. In the non-mobile group, the relative abundances of reported and novel variants did not differ significantly, whereas in the mobile group they were significantly different (Wilcoxon rank-sum test, *p* = 0.0108; Figure 6B). In addition, analysis of the dissimilarity (100 – sequence identity) between BLDB-reported sequences and β-lactamase sequences from major Ambler classes in the Han River indicated significant differences in dissimilarity distributions among Ambler classes (Kruskal-Wallis test, *p* = 0.0009; Figure 6C). Class D exhibited significantly lower dissimilarity compared with classes B and C (Kruskal-Wallis test followed by Dunn’s *post-hoc* test, *p* = 0.0048 for class B, *p* = 0.0049 for class C; Figure 6C). In addition, amino acid substitutions in the 151 unique novel β-lactamase variants were mapped to conserved motifs of the corresponding Ambler classes through pairwise alignment with their closest BLDB reference sequences. Five variants contained substitutions within or immediately adjacent to conserved β-lactamase motifs (Supplementary Table 4), suggesting that these substitutions may have functional relevance. Functional characterization, including heterologous expression, biochemical assays, and antimicrobial susceptibility testing, will be necessary to establish the functional consequences of these novel variants.

**FIGURE 6:**
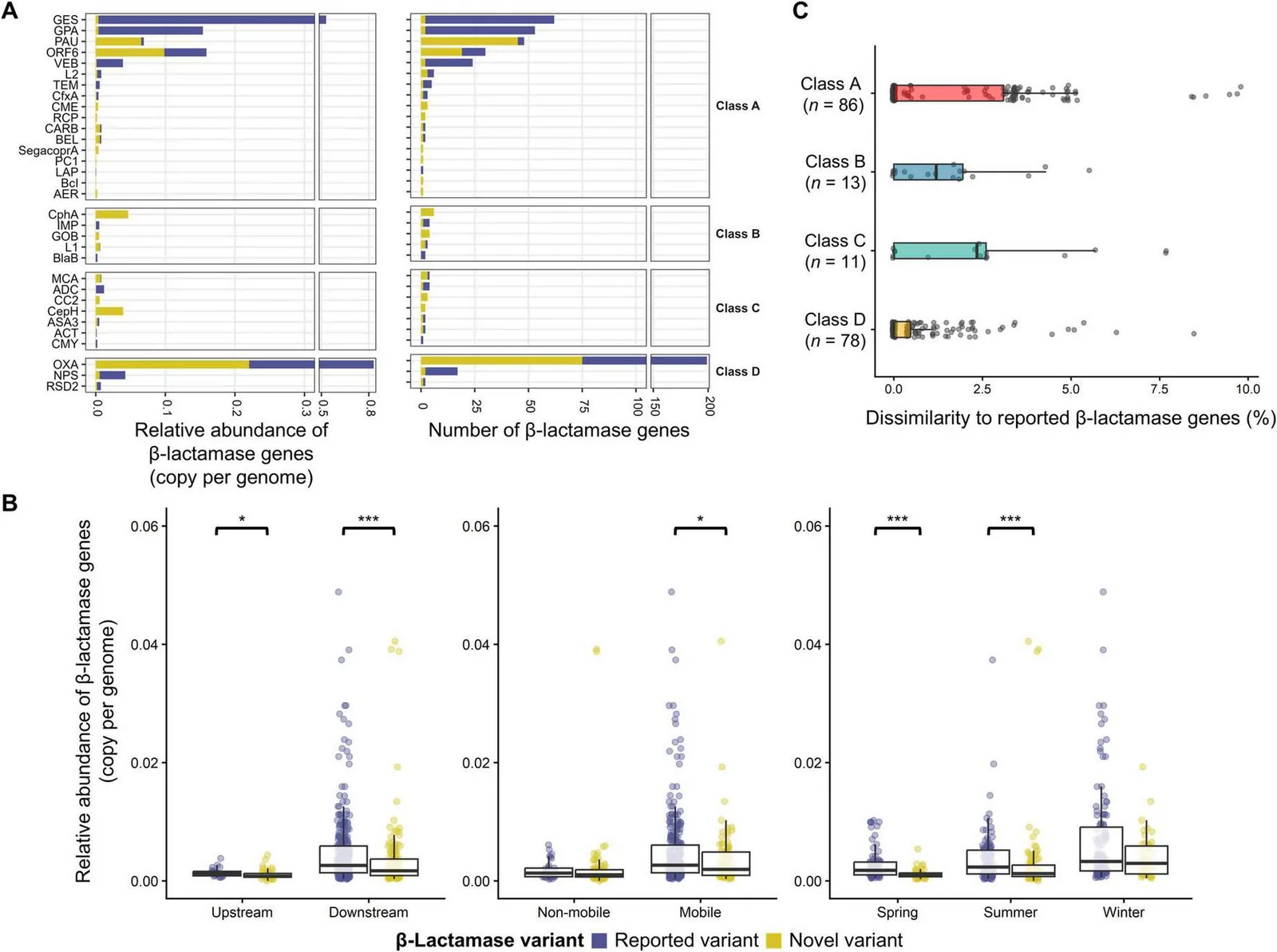
Distribution of novel β-lactamase variants. **(A)** Composition of reported and novel β-lactamase variants based on relative abundance and the number of detected β-lactamase genes. **(B)** Comparison of β-lactamase gene abundance between reported and novel variants according to geographic location, gene mobility, and season. Statistical significance is indicated by * (*p* < 0.05) and *** (*p* < 0.001). **(C)** Distribution of sequence dissimilarity (100–sequence identity) of β-lactamase genes relative to BLDB reference sequences across Ambler classes.

### 3.6 Interconnectedness of β-lactamase genes and their hosts

A bipartite network analysis of β-lactamase gene clusters revealed patterns of resistance genes shared among bacterial hosts taxonomically assigned at the metagenomic contig level (Figure 7 and Supplementary Table 5). The largest network module, located at the center of the graph, comprised several major bacterial genera, including *Aeromonas*, *Acinetobacter*, *Enterobacter*, *Klebsiella*, *Pseudomonas*, and *Stenotrophomonas*. Within this module, the genus *Aeromonas* harbored the highest diversity of β-lactamase genes, whereas the genera *Pseudomonas* and *Klebsiella* acted as primary hosts, with their mobile genes exhibiting high interconnectivity with multiple other taxa, suggesting that these bacterial genera play a pivotal role in mediating β-lactamase gene transmission. The most prevalent β-lactamase gene families associated with the greatest number of taxa in this module were OXA, GES and ORF6. In particular, the OXA-10_1 β-lactamase gene cluster was the most mobile, being shared among five different genera. Similarly, the GES-5_1, GPA-1_1, and ORF6-1_1 gene clusters were each associated with four genera, further indicating their high mobility. Given that all of these highly mobile β-lactamase genes belong to Ambler classes A and D, these findings underscore the importance of monitoring these specific classes and families in the Han River. In contrast to the highly connected central module, the genus *Shinella* formed a distinct network module connected exclusively with non-mobile β-lactamase genes.

**FIGURE 7:**
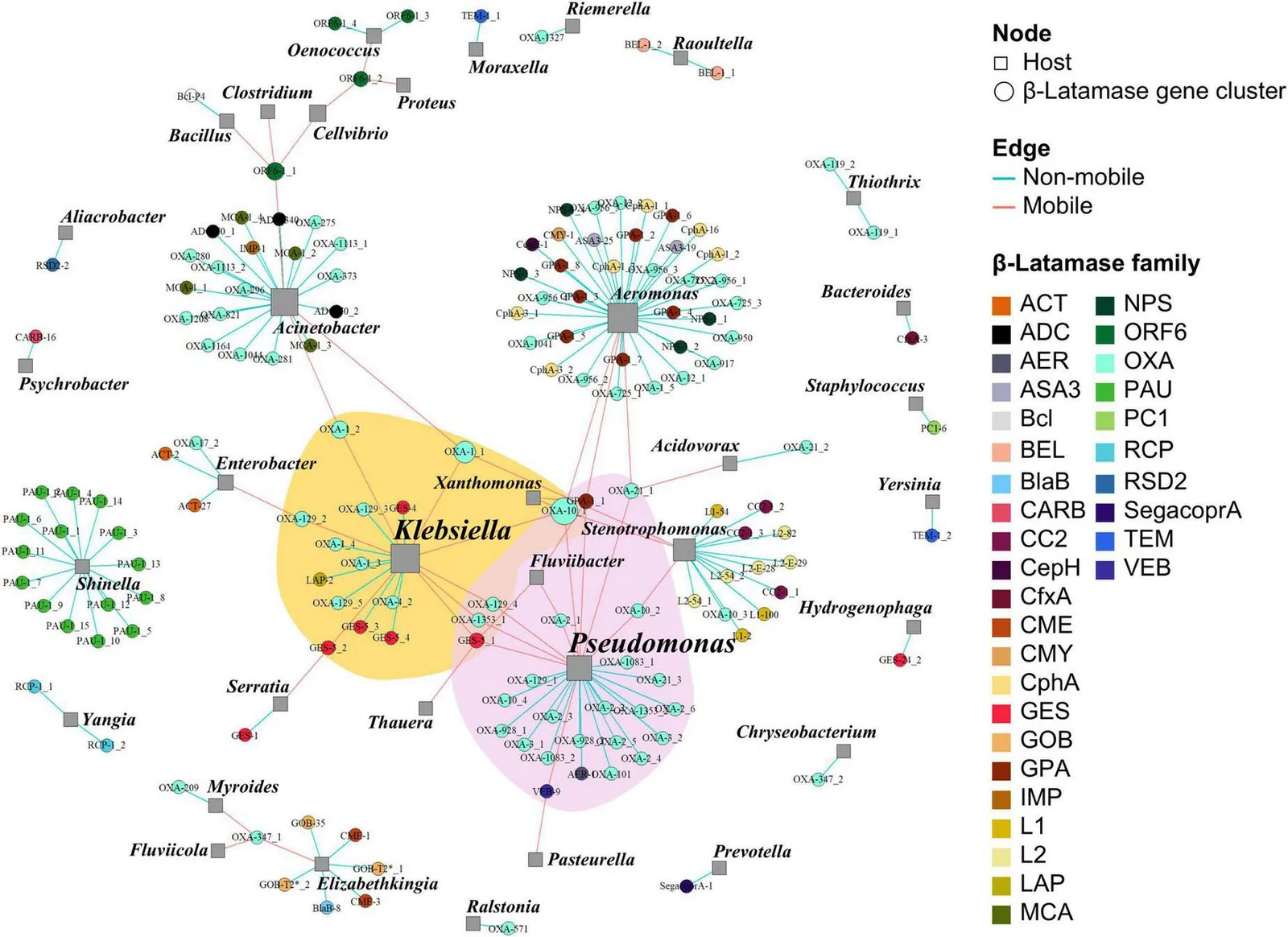
Bipartite network of β-lactamase gene clusters and their bacterial hosts. Bipartite network showing co-occurrence patterns between bacterial genera and β-lactamase gene clusters according to gene mobility.

## 4 Discussion

Given that β-lactam antibiotics are the most frequently prescribed antimicrobials in clinical settings worldwide (Wang et al., 2025), thorough monitoring and characterization of resistance to these agents are of paramount importance. Since ARGs, including those encoding β-lactamases, have been reported to spread across various environments via mobile genetic elements (Larsson and Flach, 2022), in-depth One Health research spanning multiple sectors–not limited to clinical settings–is essential (Kim and Cha, 2021; Li et al., 2025). This study addresses the limitations of clinically focused (Baleivanualala et al., 2024) or short-term surveillance (Paul et al., 2025) efforts by using a comprehensive, 5-year longitudinal metagenomic dataset to provide an in-depth evaluation of β-lactam resistance dynamics in the Han River.

Our results revealed a clear dominance of Ambler class A and D β-lactamase genes, with the class D OXA family being predominant. These findings contrast with our previous investigation of carbapenemases in the Han River bacterial isolates, where class B enzymes and the L1 family were most frequently encountered (Lee et al., 2026). This discrepancy is largely attributable to methodological differences between the two approaches. While our prior work relied on culture-dependent isolation followed by PCR targeting carbapenemase genes, the current study utilizes high-throughput shotgun metagenomics, covering the entire β-lactamase resistome. The abundance and diversity of β-lactamase genes were significantly elevated at densely populated downstream regions. This spatial pattern aligns with previous literature demonstrating that intensive anthropogenic activities serve as primary drivers for the proliferation and diversification of environmental ARGs (Gaze et al., 2011; Karkman et al., 2019; Lee et al., 2020).

Long-term trend analysis over the 5-year period highlighted that the β-lactamase dynamics in the Han River are primarily driven by mobile β-lactamase genes of classes A and D, particularly in the downstream regions. Concurrently, β-lactamase gene abundances consistently increased during the winter season. This seasonal trend is likely associated with human antibiotic consumption patterns in South Korea (Park et al., 2017), where clinical prescriptions for β-lactams typically surged during winter (Supplementary Figure 6). This is consistent with our previous findings that elevated human antibiotic consumption during winter was associated with enriched mobile ARGs, thereby driving the increase in the overall riverine resistome (unpublished data). It should also be noted that the final year of our 5-year sampling period, particularly the May and August 2020 sampling campaigns, overlapped with the COVID-19 pandemic. Pandemic-related public health measures and changes in human activities–including social distancing, altered healthcare utilization, healthcare resource reallocation, and potential changes in antibiotic consumption–may have transiently modified anthropogenic inputs to the watershed and, consequently, influenced the environmental resistome. As these potential effects were not directly evaluated in this study, they should be considered when interpreting the temporal patterns observed in 2020. In addition, the significant fold changes in the abundance of the OXA and GES families across geographical locations and seasons highlight the importance of these families in the riverine environment. Notably, as also observed in this study, the OXA and GES families are frequently associated with major pathogenic genera such as *Pseudomonas* and *Klebsiella* (Queenan and Bush, 2007; Evans and Amyes, 2014). These results underscore the ecological and public health relevance of mobile β-lactamase reservoirs in the downstream Han River.

In particular, this study identified a remarkably high proportion of novel variants. This high rate of novel variants supports the concept that the environment serves as a vast reservoir of ARGs, where the evolution of ARGs is continuously occurring (Forsberg et al., 2012; Sommer et al., 2017; Larsson and Flach, 2022). Furthermore, differences in variant composition among β-lactamase genes across geographical, seasonal, and mobility contexts suggest that mobile β-lactamase genes, which have a high potential for horizontal gene transfer, are subject to distinct evolutionary constraints and patterns of sequence conservation (San Millan et al., 2015; Nguyen et al., 2022).

In conclusion, this study provides compelling evidence that β-lactamase gene dynamics in the Han River–a major river system spanning a vast metropolitan area–are primarily driven by mobile β-lactamase genes, which facilitate their long-term dissemination and undergo β-lactamase lineage-specific diversification. These findings suggest that proactive management of β-lactamase genes in aquatic environments is a fundamental strategy for mitigating future AMR crises within the One Health framework. Ultimately, our results contribute to identifying β-lactamase gene transmission pathways between natural and clinical environments and to tracking their evolutionary trajectories.

## Data Availability

The datasets presented in this study can be found in online repositories (NCBI SRA under BioProject accession number PRJNA530373).

## Footnotes

1
https://ftp.ncbi.nlm.nih.gov/refseq/release/plasmid/
