# Supplementary material for: Long-term spatiotemporal dynamics of β-lactamase resistome in the Han River, South Korea
Source: Front Microbiol. 2026 Oct 2;17:1903212. doi: 10.3389/fmicb.2026.1903212 (PMC13672607; doi:10.3389/fmicb.2026.1903212)
Supplement: Supplementary file 1 [file Supplementary_file_1.docx]

**Supplementary material**

**Long-term spatiotemporal dynamics of β-lactamase resistome in the Han River, South Korea**

Do-Hoon Lee^1†^, Yong-Seok Kim^1†^, Dae-Wi Kim^2^, Dong-Sook Kim^3^ and Chang-Jun Cha^1*^

^1^Department Systems Biotechnology and Center for Antibiotic Resistome, Chung-Ang University, Anseong 17546, Republic of Korea

^2^Department of Life Sciences, Jeonbuk National University, Jeonju 54896, Republic of Korea

^3^Department of Health Administration, College of Health, Kongju National University, 56 Gongjudaehak-ro, Gongju-si, Chungcheongnam-do, 32588, Republic of Korea

^†^These authors contributed equally to this work

^*^Correspondence: [cjcha@cau.ac.kr](mailto:cjcha@cau.ac.kr)

Supplementary Figure 1 Locations of sampling sites in the Han River. Geographic locations are categorized as upstream (green triangles) or downstream (red circles) based on the population density at each sampling site.

Supplementary Figure 2 Distribution of β-lactamase families among bacterial hosts at the genus level.

Supplementary Figure 3 Seasonal distribution and diversity of β-lactamase genes. **(A)** β-Lactamase gene abundance across sampling sites and seasons. **(B)** Comparison of α-diversity (Shannon index) of β-lactamase genes across seasons. **(C)** Principal coordinate analysis (PCoA) of β-diversity based on the Bray–Curtis dissimilarity matrix of β-lactamase profiles across seasons.

Supplementary Figure 4 Inter-seasonal dynamics of β-lactamase genes. Inter-seasonal transition trends in total **(A)**, mobile **(B)**, and non-mobile **(C)** β-lactamase gene abundances. Linear trend lines were determined using a generalized linear model (GLM), and shaded areas represent 95% confidence intervals. The secondary y-axis on the right corresponds to the GLM-fitted trends and their confidence intervals. Statistical significance is indicated by * (*p* < 0.05), ** (*p* < 0.01), and *** (*p* < 0.001).

Supplementary Figure 5 Distribution and diversity of novel β-lactamase variants. Number of amino acid substitutions observed in novel β-lactamase variant sequences across β-lactamase gene families.

Supplementary Figure 6 β-Lactam antibiotic consumption in South Korea over the study period. Antibiotic consumption is calculated as defined daily doses per 1,000 inhabitants per day (DID).

Supplementary Table 1 Geographic information of the sampling sites

| **Site name** | **Location** | **GPS position** |
| --- | --- | --- |
| S1 | Upstream | 37°26'0.51", 128°39'27.18" |
| S2 | Upstream | 37°0'22.96", 127°59'2.4" |
| S3 | Upstream | 37°17'23.51", 127°39'5.92" |
| SB | Upstream | 37°23'46.06", 127°32'40.09" |
| S4 | Upstream | 37°29'1.61", 127°29'28.48" |
| N1 | Upstream | 38°5'14.95", 127°45'41.24" |
| N2 | Upstream | 37°50'5.35", 127°40'41.1" |
| N3 | Upstream | 37°40'28.6", 127°23'2.86" |
| H1 | Upstream | 37°32'29.75", 127°14'35.2" |
| H2 | Downstream | 37°34'31.77", 127°9'38.17" |
| H3 | Downstream | 37°32'2.06", 127°2'15.63" |
| H4 | Downstream | 37°31'31.66", 127°1'0.82" |
| H5 | Downstream | 37°33'0.27", 126°53'46.28" |
| H6 | Downstream | 37°35'44", 126°49'1.46" |
| H7 | Downstream | 37°39'6.73", 126°43'17.93" |

Supplementary Table 2 Summary statistics of generalized linear models testing temporal changes in total and Ambler class-specific β-lactamase gene abundances.

|  |  | **Estimate** | **Standard error** | **t-value** | **p-value** | **Dispersion**  **parameter** | **Null**  **deviance** | **Residual**  **deviance** | **AIC** |
| --- | --- | --- | --- | --- | --- | --- | --- | --- | --- |
| **Total** | Intercept | 2.61e−03 | 4.38e−04 | 5.968 | 4.57e−09 | 3.56e−05 | 0.0183 | 0.0177 | −3698.4 |
|  | Slope | 6.04e−05 | 1.48e−05 | 4.077 | 5.32e−05 |  |  |  |  |
| **Class A** | Intercept | 2.12e−03 | 6.18e−04 | 3.438 | 0.000689 | 3.02e−05 | 0.0078 | 0.0073 | −1850.7 |
|  | Slope | 7.62e−05 | 1.99e−05 | 3.828 | 0.000164 |  |  |  |  |
| **Class B** | Intercept | 5.55e−03 | 2.95e−03 | 1.879 | 0.0774 | 7.60e−05 | 0.0014 | 0.0013 | −122.4 |
|  | Slope | −1.21e−04 | 1.22e−04 | −0.99 | 0.3359 |  |  |  |  |
| **Class C** | Intercept | 4.15e−03 | 2.43e−03 | 1.704 | 0.108 | 8.34e−05 | 0.0013 | 0.0013 | −114.09 |
|  | Slope | −1.38e−05 | 1.84e−04 | −0.075 | 0.941 |  |  |  |  |
| **Class D** | Intercept | 2.57e−03 | 6.48e−04 | 3.96 | 0.000102 | 3.49e−05 | 0.0078 | 0.0075 | −1614.4 |
|  | Slope | 6.41e−05 | 2.22e−05 | 2.894 | 0.004198 |  |  |  |  |

Supplementary Table 3 Summary statistics of generalized linear models testing inter-seasonal differences in β-lactamase gene abundances.

|  |  |  | **Estimate** | **Standard**  **error** | **t-value** | **p-value** | **Dispersion**  **parameter** | **Null**  **deviance** | **Residual**  **deviance** | **AIC** |
| --- | --- | --- | --- | --- | --- | --- | --- | --- | --- | --- |
| **Total** | **Winter → Spring** | Intercept | 0.005982 | 0.000489 | 12.227 | < 2e−16 | 4.24e−05 | 0.0109 | 0.010337 | −1774.9 |
|  |  | Slope | −0.00115 | 0.000308 | −3.731 | 0.000237 |  |  |  |  |
|  | **Spring → Summer** | Intercept | 0.002181 | 0.000428 | 5.093 | 6.01e−07 | 2.27e−05 | 0.0074 | 0.007296 | −2532.8 |
|  |  | Slope | 0.000422 | 0.000182 | 2.321 | 0.0209 |  |  |  |  |
|  | **Summer → Winter** | Intercept | 0.002902 | 0.000555 | 5.228 | 3.08e−07 | 4.59e−05 | 0.0156 | 0.014874 | −2327.2 |
|  |  | Slope | 0.000513 | 0.000126 | 4.088 | 5.48e−05 |  |  |  |  |
| **Mobile** | **Winter → Spring** | Intercept | 0.006737 | 0.000572 | 11.775 | < 2e−16 | 4.88e−05 | 0.0102 | 0.009706 | −1421.2 |
|  |  | Slope | −0.00123 | 0.000375 | −3.269 | 0.00127 |  |  |  |  |
|  | **Spring → Summer** | Intercept | 0.002513 | 0.000449 | 5.596 | 6.62e−08 | 1.98e−05 | 0.0044 | 0.00425 | −1730.6 |
|  |  | Slope | 0.000507 | 0.000202 | 2.508 | 0.0129 |  |  |  |  |
|  | **Summer → Winter** | Intercept | 0.003182 | 0.000814 | 3.911 | 0.000122 | 5.10e−05 | 0.0121 | 0.011421 | −1588.4 |
|  |  | Slope | 0.000592 | 0.000167 | 3.547 | 0.000475 |  |  |  |  |
| **Non-**  **mobile** | **Winter → Spring** | Intercept | 1.86e−03 | 2.00e−04 | 9.334 | 6.60e−11 | 7.57e−07 | 3.36e−05 | 2.58e−05 | −401.26 |
|  |  | Slope | −3.11e−04 | 9.69e−05 | −3.211 | 0.00289 |  |  |  |  |
|  | **Spring → Summer** | Intercept | 0.000926 | 0.001034 | 0.896 | 0.372 | 2.78e−05 | 0.0029 | 0.0029 | −807.15 |
|  |  | Slope | 0.000549 | 0.000397 | 1.383 | 0.17 |  |  |  |  |
|  | **Summer → Winter** | Intercept | 0.002602 | 0.000672 | 3.872 | 0.000205 | 3.25e−05 | 0.0029 | 0.002894 | −678.15 |
|  |  | Slope | −0.00012 | 0.000245 | −0.502 | 0.61671 |  |  |  |  |

Supplementary Table 4 Amino acid substitutions within or adjacent to conserved motifs of β-lactamases in novel variants

| **Sequence ID** | **Ambler class** | **Closest β-lactamase from BLDB** | **Motif pattern** | **Motif sequence of reported variants** | **Motif sequence of novel variants** | **Amino acid substitution** |
| --- | --- | --- | --- | --- | --- | --- |
| 1605_H6_5-H6-2_0698774_1 | A | VEB-14 | ExxxN | EEQMH | KEQMH | E166K |
| 1809_H1_contig-149_10450_5 |  | CME-1 |  | EEEMH | EEEMS | H170S |
| 1908_N3_contig-149_327054_1 |  | CME-3 |  | EEEMH | EEEMS |  |
| 1608_H3_8-H3_2_0351200_1 |  | PAU-1 | SxxK | SSFK | SPFK | S71P |
| 1902_H6_contig-149_332528_1 | B1 | IMP-1 | HxHxD | HFHSD | PFHSD | H116D |

Supplementary Table 5 Network topology metrics of the bipartite network of bacterial genera and β-lactamase gene clusters. Degree and betweenness centrality of nodes are shown for nodes with degree ≥ 2. Nodes with degree = 1 (*n* = 155) were excluded, as such nodes have a betweenness centrality of 0 by definition. The overall network modularity, calculated using Beckett’s algorithm, was Q = 0.717.

| **Node** | **Type** | **Degree**  **centrality** | **Betweenness**  **centrality** |
| --- | --- | --- | --- |
| *Aeromonas* | Bacteria | 38 | 4019.12 |
| *Klebsiella* | Bacteria | 17 | 3754.48 |
| *Acinetobacter* | Bacteria | 22 | 3323.98 |
| *Pseudomonas* | Bacteria | 26 | 2777.53 |
| *Stenotrophomonas* | Bacteria | 15 | 2101.14 |
| *Cellvibrio* | Bacteria | 2 | 630 |
| *Enterobacter* | Bacteria | 4 | 387 |
| *Oenococcus* | Bacteria | 3 | 259 |
| *Acidovorax* | Bacteria | 2 | 130 |
| *Serratia* | Bacteria | 2 | 130 |
| *Bacillus* | Bacteria | 2 | 130 |
| *Shinella* | Bacteria | 15 | 105 |
| *Fluviibacter* | Bacteria | 3 | 41.81 |
| *Elizabethkingia* | Bacteria | 7 | 39 |
| *Xanthomonas* | Bacteria | 2 | 12.95 |
| *Myroides* | Bacteria | 2 | 9 |
| *Raoultella* | Bacteria | 2 | 1 |
| *Yangia* | Bacteria | 2 | 1 |
| *Thiothrix* | Bacteria | 2 | 1 |
| OXA-10_1 | ARG | 5 | 3412.08 |
| OXA-1_1 | ARG | 3 | 2119.47 |
| ORF6-1_1 | ARG | 4 | 1118 |
| OXA-1_2 | ARG | 2 | 1023.78 |
| OXA-21_1 | ARG | 3 | 548.4 |
| ORF6-1_2 | ARG | 3 | 511 |
| OXA-129_2 | ARG | 2 | 508 |
| GES-5_1 | ARG | 4 | 471.54 |
| GPA-1_1 | ARG | 4 | 397.67 |
| OXA-129_4 | ARG | 2 | 262.18 |
| OXA-1353_1 | ARG | 2 | 262.18 |
| GES-5_2 | ARG | 2 | 258 |
| OXA-10_2 | ARG | 2 | 244.68 |
| VEB-9 | ARG | 2 | 130 |
| OXA-347_1 | ARG | 3 | 23 |
| OXA-2_1 | ARG | 2 | 11.02 |
